# Socio-Economic Disparities in the Burden of Seasonal Influenza: The Effect of Social and Material Deprivation on Rates of Influenza Infection

**DOI:** 10.1371/journal.pone.0017207

**Published:** 2011-02-17

**Authors:** Katia M. Charland, John S. Brownstein, Aman Verma, Stephanie Brien, David L. Buckeridge

**Affiliations:** 1 Children's Hospital Informatics Program, Children's Hospital Boston, Boston, Massachusetts, United States of America; 2 Department of Pediatrics, Harvard Medical School, Boston, Massachusetts, United States of America; 3 McGill Clinical and Health Informatics, McGill University, Montreal, Canada; 4 Department of Epidemiology, Biostatistics and Occupational Health, McGill University, Montreal, Canada; 5 Office of Surveillance and Epidemiology, Montreal Public Health Department, Montreal, Canada; Aga Khan University, Pakistan

## Abstract

**Background:**

There is little empirical evidence in support of a relationship between rates of influenza infection and level of material deprivation (i.e., lack of access to goods and services) and social deprivation (i.e. lack of social cohesion and support).

**Method:**

Using validated population-level indices of material and social deprivation and medical billing claims for outpatient clinic and emergency department visits for influenza from 1996 to 2006, we assessed the relationship between neighbourhood rates of influenza and neighbourhood levels of deprivation using Bayesian ecological regression models. Then, by pooling data from neighbourhoods in the top decile (i.e., most deprived) and the bottom decile, we compared rates in the most deprived populations to the least deprived populations using age- and sex-standardized rate ratios.

**Results:**

Deprivation scores ranged from one to five with five representing the highest level of deprivation. We found a 21% reduction in rates for every 1 unit increase in social deprivation score (rate ratio [RR] 0.79, 95% Credible Interval [CrI] 0.66, 0.97). There was little evidence of a meaningful linear relationship with material deprivation (RR 1.06, 95% CrI 0.93, 1.24). However, relative to neighbourhoods with deprivation scores in the bottom decile, those in the top decile (i.e., most materially deprived) had substantially higher rates (RR 2.02, 95% Confidence Interval 1.99, 2.05).

**Conclusion:**

Though it is hypothesized that social and material deprivation increase risk of acute respiratory infection, we found decreasing healthcare utilization rates for influenza with increasing social deprivation. This finding may be explained by the fewer social contacts and, thus, fewer influenza exposure opportunities of the socially deprived. Though there was no evidence of a linear relationship with material deprivation, when comparing the least to the most materially deprived populations, we observed higher rates in the most materially deprived populations.

## Introduction

Defining subpopulations that initiate and promote influenza epidemics can help to guide the strategic distribution of prevention and control efforts. To date, researchers have focused primarily on the effect of age and have found that the paediatric population plays an important role in transmission [1], [2], [3]. Children tend to be infected earlier in the season and due to their more immature immune systems and more extensive contact networks, may spread the virus more readily than older age groups [3]. Socio-economically deprived populations may also experience higher rates of acute respiratory infection. However, research in this area is largely dominated by studies of hospitalizations and mortality [4], [5], [6] providing evidence that rates of severe illness and not necessarily rates of infection are elevated in this population [4], [5], [6], [7], [8], [9], [10].

Socio-economic deprivation is a broad term that encompasses various aspects of socio-economic vulnerability. A number of composite measures have been developed as markers of deprivation. Townsend (1987) described two distinct forms of deprivation; *material deprivation*, a measure of access to ‘goods and conveniences’ and *social deprivation*, representing social cohesion, cooperation and support [11]. A few studies have linked *material* deprivation to rates of hospitalization and mortality due to respiratory infection [4], [5], [6], [12]. Changes in rates of respiratory illness in relation to social deprivation have not been studied as extensively, but it has been suggested that the stress resulting from social deprivation as well as its effects on personal habits and self-esteem may predispose individuals to infection [13], [14], [15]. Results from studies examining the impact of certain aspects of social deprivation (e.g. social support) on hospitalizations for acute respiratory illness are mixed, finding either a positive relationship or no relationship with admission rates [6] [9].

In this study, we assessed the relationship between material and social deprivation and rates of emergency department and outpatient clinic (ED/OC) utilization for influenza in all 111 neighbourhoods of Montreal, Quebec over the period 1996 to 2006. In doing so, we explore whether directing public health interventions towards socio-economically deprived neighbourhoods could mitigate population-wide morbidity and mortality rates.

## Methods

### Geographic Partition of the Island of Montreal

The department of public health (*Direction de la santé publique*) and the health and social services agency of Montreal (*Agence de la santé et des services sociaux de Montréal*), in collaboration with local communities, have created a neighbourhood partition of Montreal that preserves within-neighbourhood homogeneity with respect to socio-demographic factors (http://www.cmis.mtl.rtss.qc.ca/fr/atlas/creer_carte/details_creer_carte.html). These neighbourhoods were formed by aggregating census tracts, the smallest administrative region for which we had ED/OC utilization data. Although the finest partition is generally preferred to minimize ecological bias, there were several advantages to using a neighbourhood partition. Census tracts contain only 4000 individuals on average, thus census tract-level risk estimates lacked precision. Basing the analysis on the neighbourhood partition provided a balance between precision of risk estimates and reduction of ecological bias. In addition, the analyses were facilitated by the consistency of the neighbourhood boundaries throughout the study period.

### Census data and billing claims for visits to outpatient clinics and emergency departments

The *Régie de l'assurance maladie du Québec* (RAMQ) is the government body that provides health insurance to 99% of the residents of the province of Quebec, Canada. For our study, we obtained billing claim records for visits to outpatient clinics and emergency departments, by residents of Montreal, from 1996 to 2006 for influenza-like illness. Each record provided data on patient age, sex, census tract of residence, date of visit, and an *International Classification of Diseases, 9^th^ Revision* (ICD-9) diagnostic code. Sets of ICD-9 codes were used to define a visit for influenza. It has been shown that influenza (487) codes tend to have high specificity but low sensitivity for identifying influenza cases [16], [17], so we analysed the data using two definitions of influenza, one with only influenza-specific ICD-9 codes (487) and another with influenza and pneumonia ICD-9 codes (486 and 487). An influenza season was defined as the 40^th^ CDC week of one year to the 39^th^ CDC week of the following year. To more closely measure rates of influenza rather than rates of utilization for influenza, we counted only one visit per person per influenza season. We estimated at-risk person years for each age-sex stratum using data from the 1996, 2001 and 2006 censuses, approximating population sizes in inter-censal years by linear interpolation. Age groups were 0 to 4, 5 to 9, 10 to 14, 15 to 19, 20 to 39, 40 to 64 and 65+ years. Ethics approval was granted by the Institutional Review Board of McGill University's Faculty of Medicine.

### Material and Social Deprivation Indices

Pampalon and Raymond (2000) constructed indices of material and social deprivation for the province of Quebec [18]. Each index is composed of three census variables. For material deprivation, the variables are proportion of persons lacking a high school diploma, employment to population ratio, and average income. The census variables for social deprivation are proportion of persons living alone, the proportion of persons separated, divorced or widowed, and the proportion of single parent families. Deprivation was measured for all *dissemination areas (DA)* in the region and quintiles of deprivation were formed with the value of 1 representing the lowest levels of deprivation. As each neighbourhood geographical unit is comprised of several DA, we calculated a neighbourhood deprivation score by averaging the deprivation quintile values of the DA contained within the boundaries of the neighbourhood, using DA population sizes as weights.

### Statistical Analyses

#### Ecological Regressions

Preliminary analyses consisted of estimating correlations between deprivation scores and the neighbourhood standardized morbidity ratios (SMR). We also constructed choropleth maps of the SMR and deprivation scores. Assuming that the number of healthcare visits for influenza for each neighbourhood was Poisson distributed, we used Bayesian hierarchical models to examine the strength of the linear association between the log of the relative risk and each measure of deprivation. In all models, we accounted for spatial autocorrelation as failing to do so could invalidate inferences [19]. We considered four models, one with social deprivation as the predictor, another with material deprivation, the third with both social and material deprivation, and the fourth with social and material deprivation main effects and their interaction. Covariates were centred to improve convergence of the Monte Carlo Markov Chains.

#### Contrasting rates in the most and least deprived neighbourhoods

We examined whether the most deprived populations had significantly different rates than the least deprived populations. To do this, we identified the neighbourhoods with deprivation scores in the top and bottom decile (i.e. the most and least deprived, respectively) and then pooled their counts of influenza-related visits and their at-risk person time. Age and sex-standardized rate ratios were estimated using the least deprived population as the reference group. We assessed the sensitivity of our results to the percentage of neighbourhoods included in the extremes by also comparing the top and bottom 5% and 15%. Analyses were conducted with R and WinBUGS 1.4 software [20], [21]. Maps were constructed using maps2WinBUGS version 1.6.

## Results

**Figure 1:**
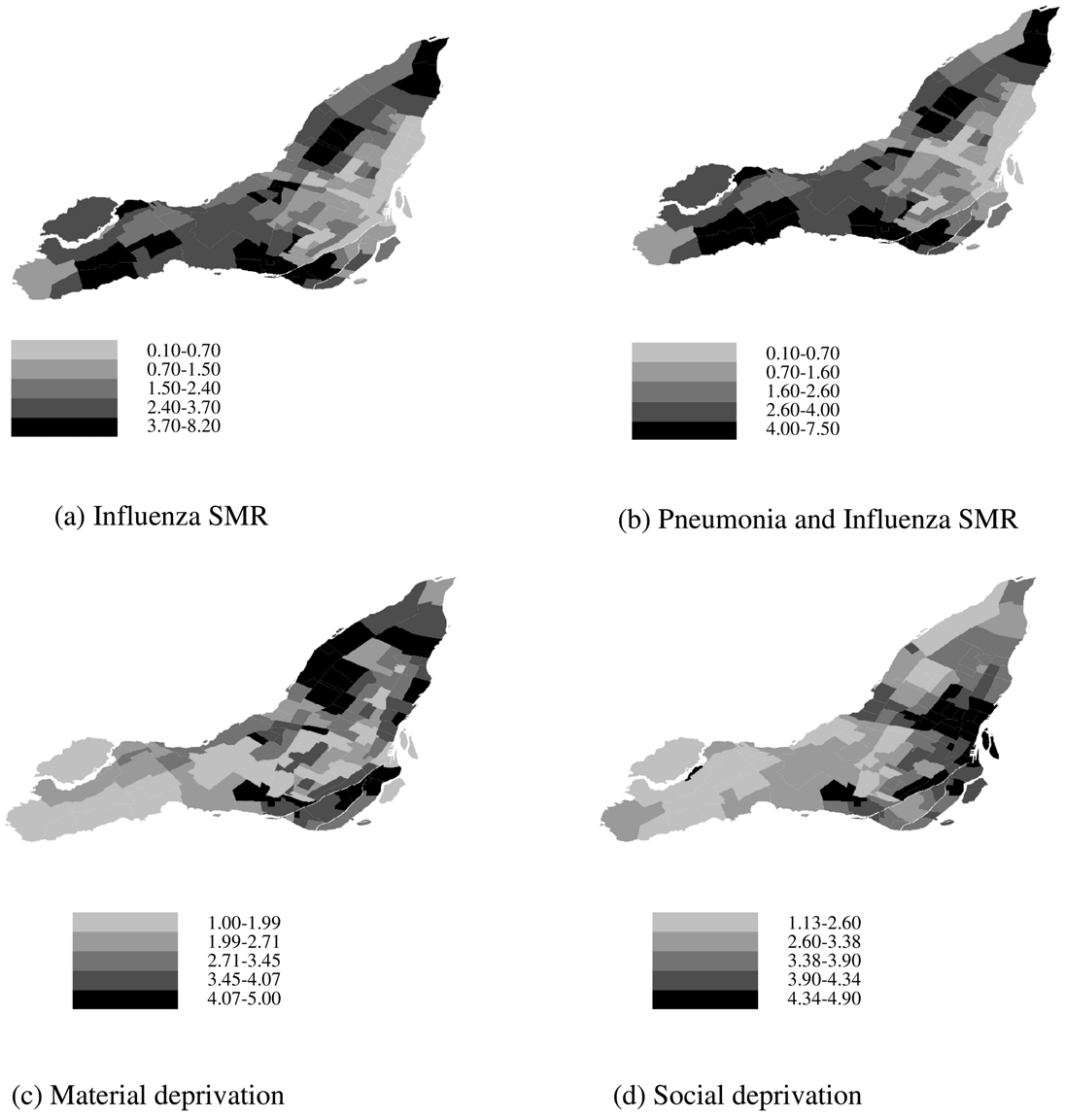
Choropleth maps of neighborhood standardized morbidity ratios and deprivation scores. Standardized morbidity ratio for influenza (a), standardized morbidity ratio for pneumonia and influenza (b), Material deprivation (c), Social Deprivation (d).

The correlation between the neighbourhood-level log standardized morbidity ratio (SMR) and the material and social deprivation score was 0.11 and −0.48, respectively. This finding is reflected in the choropleth maps (Figure 1), where areas with greater levels of social deprivation tended to have smaller SMRs. The results of the ecological regression indicated an average decrease in utilization rates by approximately 21% for every 1 unit increase in social deprivation score (Table 1). There did not appear to be a meaningful linear relationship with material deprivation (Table 1), nor was there evidence of an important interaction between social and material deprivation (regression coefficient −0.066, 95% CI −0.17 to 0.14). Results using the Pneumonia and Influenza definition were consistent with that of the Influenza definition (Table 1).

**Table 1:** Rate Ratio of emergency department and outpatient clinic visits given a one unit change in deprivation score.

*Model*	*Influenza*	*Pneumonia and Influenza*
*Social*	0.79 (0.66, 0.97)	0.78 (0.66, 0.92)
*Material*	1.06 (0.93, 1.24)	1.01 (0.89,1.15)
*Social+Material*	*Social*: 0.81 (0.65, 1.03)	*Social*: 0.79 (0.66, 0.99)
	*Material*: 1.06 (0.94, 1.22)	*Material*: 1.00 (0.86, 1.14)

**Figure 2:**
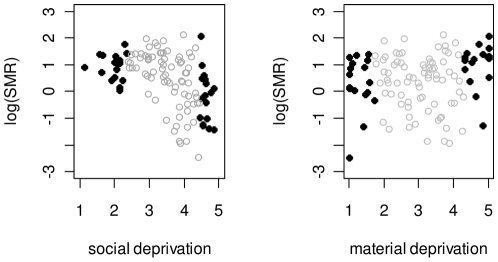
Neighbourhood log standardized morbidity ratio for influenza versus deprivation score with neighbourhoods in the top and bottom 15% in black.

Plots of the neighbourhood log SMR versus deprivation score are shown in Figure 2. The rate of utilization for influenza among populations living in the most materially deprived neighbourhoods (top decile) were 102% higher than those living in the least materially deprived neighbourhoods (Table 2). When we excluded neighbourhoods that had both high or both low material and social deprivation scores, we found an even greater disparity in rates (rate ratio [RR] 4.65, 95% Confidence Interval [CI] 4.55 to 4.76). In comparing the most to the least socially deprived neighbourhoods we found that the most socially deprived populations had approximately 79% lower utilization rates for influenza and 81% lower rates for pneumonia and influenza (Table 2). When neighbourhoods with deprivation scores in the top and bottom 5% and 15% were analysed, we found similar patterns of elevated risk in the materially deprived populations and decreased risk in the socially deprived populations were observed (Table 2).

**Table 2:** Adjusted rate ratios and 95% confidence intervals for comparison of most deprived to least deprived populations using influenza definition.

*Percent*	*Form of Deprivation*	*Influenza Rate Ratio*	*Pneumonia and Influenza Rate Ratio*
*5%*	*Social*	0.211 (0.205, 0.216)	0.185 (0.181, 0.189)
	*Material*	2.34 (2.28, 2.41)	2.20 (2.15, 2.25)
*10%* §	*Social*	0.219 (0.215, 0.223)	0.193 (0.190, 0.196)
	*Material*	2.02 (1.99, 2.05)	1.91 (1.89, 1.94)
*15%*	*Social*	0.244 (0.240, 0.247)	0.213 (0.210, 0.215)
	*Material*	1.92 (1.90, 1.95)	1.80 (1.78, 1.82)

§ comparison using pooled data from 11 most and 11 least deprived neighbourhoods.

## Discussion

Contrary to the hypothesized effect of risk elevation in the socially deprived, we observed decreasing rates of ED/OC utilization for influenza with increasing social deprivation. Comparing the risk in the 10% most and least socially deprived neighbourhoods, we found a pronounced ‘protective’ effect of social deprivation. Unlike studies of hospitalization/mortality rates and material deprivation, we did not find an increasing gradient in utilization rates with greater levels of material deprivation. However, as compared to the least materially deprived neighbourhoods, rates were considerably elevated in the most materially deprived neighbourhoods.

Though the negative association with social deprivation may be interpreted as the result of an overall underutilization of health services by the more socially deprived, Philibert et al (2007) found that this was not the case for a large downtown Montreal clinic in 2000–2002, a time period centred at the mid-point of our study period [22]. Thus an alternative explanation of our finding may lie in the fact that socially deprived groups have fewer social contacts and thus have fewer opportunities to become exposed to an infected individual [23]. One of the variables contributing to the social deprivation index is the proportion of the population living alone. There is evidence that indoor crowding facilitates influenza transmission [7], [24].

Findings from previous studies assessing the relationship between social deprivation and hospitalizations/mortality due to respiratory illness are mixed [6],[9]. Crighton et al (2007) conducted an ecological study in the province of Ontario, Canada, and did not find a positive association between county hospitalization rates for influenza and pneumonia and percentage of surveyed individuals reporting low levels of *social support* and *social involvement* [6]. In contrast, Jordan et al (2008) found higher rates of hospital admissions for respiratory disease (either acute illness or worsening of chronic disease) among more socially isolated elderly [9]. However, the interpretation of this finding is not straightforward. Though the socially isolated elderly may experience more severe outcomes and even a greater incidence of infection, physicians may also be more inclined to admit patients that, due to a lack of social support, will not receive care if sent home.

Ecological studies in the United Kingdom have found a relationship between an index of material deprivation and rates of hospitalizations and mortality from respiratory illnesses [4], [5]. A similar finding is reported by Crighton et al (2007) in their ecological study using data from Ontario, Canada, in which they discovered high rates of hospitalizations for pneumonia and influenza in counties where a high proportion of the population did not possess a high school diploma [6]. The frequency of occurrence of influenza/cold symptoms may also be related to material deprivation. Stone et al (2010) found through a telephone survey that the incidence of individuals reporting headache, pain or influenza/cold symptoms increased as level of educational attainment and income decreased [25].

A limitation of our study is our failure to account for ‘silent cases’, i.e. those that were sick but did not seek care. However, there is no evidence suggesting that rates of silent cases should vary by neighbourhood in an urban setting such as Montreal where residents have universal access to essential medical services [22]. Thus it is likely that the spatial variation of utilization rates for influenza approximates the spatial variation in rates of influenza infection. We were also limited by a case definition based on ICD-9 codes which may not identify all visits to ED/OCs for influenza infection. Nevertheless, we considered two influenza case definitions and results were consistent.

Ecological bias may prevent an extrapolation of our findings to the individual. However, for the purpose of designing effective public health strategies, it may be more feasible to direct interventions to neighbourhoods. In this case, inference at the level of the neighbourhood, and not the individual, is most pertinent. Even so, both the ecological and individualistic fallacies can be dealt with to some extent through an analysis of individual level outcomes that incorporates both individual and area-level deprivation data [26], [27], [28]. In our future work we will assess the relationship between ED/OC utilization rates for influenza and both individual-level and neighbourhood-level factors.

### Conclusion

Though some studies have found an association between socio-economic deprivation and acute respiratory illness, we did not find evidence of increasing risk of influenza with increasing material deprivation. We did note, however, a disparity in the burden of influenza when comparing the extremes of material deprivation. Though social deprivation is a hypothesized risk factor for disease, we observed lower rates of visits to the emergency department and outpatient clinics for influenza from more socially deprived populations, a finding which may be explained by reduced infection exposure opportunities.
